# Editorial: Bone marrow adiposity - contributions to bone, aging and beyond

**DOI:** 10.3389/fendo.2023.1144163

**Published:** 2023-02-02

**Authors:** Jason A. Horton, Sarah Beck-Cormier, Andre J. van Wijnen

**Affiliations:** ^1^ Deparment of Orthopedic Surgery, State University of New York (SUNY) Upstate Medical University, Syracuse, NY, United States; ^2^ Nantes Université, The National Center for Scientific Research (CNRS), The National Institute of Health and Medical Research (INSERM), l’institut du thorax, Nantes, France; ^3^ Department of Biochemistry, University of Vermont, Burlington, VT, United States

**Keywords:** bone marrow adipose tissue (BMAT), osteoporosis, diabetes, adipocyte biology, bone biology and physiology

## Introduction

Bone marrow adipose tissue (BMAT) plays a complex role in regulating various biological processes, including metabolism, endocrine and immune functions, hematopoeisis and skeletal homeostasis. However, our understanding of the physiological and pathological roles of BMAT remains limited. For this Special Issue on BMAT, we selected 14 articles that provide a comprehensive overview of BMAT, including 4 reviews, 8 original research papers and 2 conference reports. The articles cover a range of topics related to the biology of BMAT in health and disease, and include validated methodologies and overviews of concepts relevant to researchers in the field.

## Realizing the vision of the International Bone Marrow Adiposity Society

The International Bone Marrow Adiposity Society (BMAS) has held 7 annual meetings since 2015, with the sixth meeting held virtually in 2020 due to the COVID-19 pandemic (Scheller et al.). The meeting was attended by nearly 200 people from 18 countries and featured presentations, networking opportunities and career development. The BMAS also held its inaugural Summer School in 2021, which was aimed at early-career researchers and featured lectures, workshops and career development sessions (Labella et al.). The Biobanking Working Group of the BMAS has developed best practices for preparing, preserving and distributing biomaterials and related cells and tissues as biospecimens for research, including protocols and guidelines for ethical, legal and social issues (Lucas et al.). These best practices aim to ensure the rigor and collaborations in the field of BMAT research.

## Origins and phenotypes of BMAT in development and aging

This Special Issue contains two informative studies that assess lineage commitment of mesenchymal stem cells. Matsushita et al. reviewed data from single-cell RNA sequencing and lineage tracing studies to build a framework for understanding how bipotent bone marrow stromal cell (BMSC) progenitors differentiate along adipocytic or osteogenic trajectories. This study discussed the inverse relationship between marrow adiposity and bone formation rate and the authors propose that these lineages may be plastic and dynamic, rather than mutually exclusive and static. Evidence of this was presented by Lee et al. whom used a lineage tracing approach the relationship bone marrow adipocytes (BMAd) and osteoblasts. The authors examined mice with a fluorescent osteocyte-specific lineage tracer (i.e., tamoxifen-induced DMP1Cre-driven GFP reporter). In these mice, exposure to the thiazolidinedione (TZD) drug rosiglitazone, which activates Peroxisome-Proliferator Activated Receptor Gamma (PPARγ/PPARG), leads to an expansion of BMAT that is positive for perilipin 1 (PLIN1), a definitive adipocyte marker. However, a small fraction (~5%) of these cells also expressed GFP, which was not observed in control mice, suggesting that osteocytes can convert into adipocytes. The osteoanabolic drug Romosozumab slightly reduced BMAT expansion and eliminated the GFP+/PLIN1+ population. These findings may have implications for the management of co-occurring skeletal and metabolic conditions. Future studies will need to refine how control of cell fate determination of mesenchymal stem cells relates to trans-differentiation of phenotypically committed cells.

Tratwal et al. used single-cell raman microspectroscopy to characterize the lipid saturation profile of murine BMSC-like OP9 cells under induced and spontaneous adipogenic differentiation conditions and compared it to human primary samples with predominantly hematopoietic marrow (iliac crest) or fatty marrow (femoral head). They found that the lipid profile of spontaneously differentiated OP9 cells closely resembled that of hematopoiesis-supportive regulated marrow adipocytes (rMAd), while chemically-induced differentiation promoted a constitutive marrow adipocyte (cMAd)-like phenotype that did not effectively support hematopoiesis. These results offer a new way to differentiate rMAd and cMAd that may be useful for further study of their development and functional differences.

Aaron et al. reviewed the effects of BMAT expansion on bone homeostasis, immune and endocrine function, BMSC biology and bone regeneration during aging. They discuss how BMAT can either support or suppress the growth and differentiation of hematopoietic stem cells (HSCs) and can induce pro-inflammatory cytokines and ROS that contribute to senescence in BMSCs and impair their function. The review suggests potential strategies, such as senolytics, miRNAs and antioxidants, to improve the function of BMSCs and reduce the negative effects of aging on the bone marrow microenvironment, paving the way for further research.

Identification of new regulators for bone and adipose tissues is important for the ageing population being more susceptible to bone loss and fat gain. Considering the deleterious effect of the PiT2 sodium-phosphate co-transporter (SLC20A2) deficiency in bone, Frangi et al. addressed whether the BMAT could also be affected in PiT2/Scl20a2 knockout mice. They found that PiT2-deficient young mice have high level of BMAT, but that this volume does not increase into adulthood, leading to lower BMAT volume in older PiT2-deficient mice. However, the absence of PiT2 did not prevent an increase in BMAT volume in a model of bone loss induced by ovariectomy. PiT2-deficient mice did not have differences in serum phosphate levels or key markers of phosphate regulation, suggesting that the observed defects in BMAT were not related to serum phosphate levels. Their findings suggest that PiT2 plays a role in the maintenance of both bone and BMAT, probably independently of systemic phosphate homeostasis although the cellular and molecular mechanisms remain to be identified.

## Role and responses of BMAT in pathophysiology

Ali et al. examined how conditions such as hormone deficiency, obesity and type 2 diabetes can contribute to the development of osteoporosis by increasing the negative impact of BMAT on bone health. They discussed several mechanisms involved in these effects, including transcription factors, DNA damage and the production of inflammatory cytokines that alter the fate of BMSCs. The authors also discussed lifestyle modifications, including diet and exercise, as strategies to improve bone parameters and modulate BMAT in patients with metabolic diseases. They noted that antiresorptive drugs may not be effective in all patients with metabolic diseases and discussed research suggesting that Denosumab may improve insulin sensitivity, bone formation and muscle strength in some cases, but emphasized that need for further research is needed to understand how these co-morbid conditions may be best managed.

Sollmann et al. used advanced image analysis techniques to improve the accuracy of fracture risk prediction in patients with osteoporosis. The authors suggest that incorporating texture analysis of CT and MRI T2* data based on Proton Density Fat fraction (PDFF) may be more effective at predicting and differentiating between patients with and without osteoporotic vertebral fractures compared to bone mineral density (BMD) and PDFF alone.

Janus kinase inhibitors such as Tofacitinib are used to control systemic inflammatory disorders such as rheumatoid arthritis (RA), and may have beneficial effects on bone, but their effect on BMAds is unknown. Letarouilly et al. investigated the effects Tofacitinib, on BMAd and osteoblasts derived from human BMSCs and in RA patients treated with Tofacitinib. Tofacitinib increased BMAd differentiation and decreased osteoblast differentiation of primary cells under non-inflammatory conditions. In RA patients, Tofacitinb increased lumbar spine PDFF and had no effect on BMD or body composition over a 6-month period. These results suggest that Tofacitinib has a stimulatory effect on BMAd commitment and differentiation, which may not support its beneficial effects on the bone microenvironment. Further studies are needed to determine whether this is a class effect of Janus kinase inhibitors or specific to Tofacitinib.

Dello Spedale Venti et al. compared the histopathologic characteristics of BMAds in patients with bone metastasis and myeloproliferative neoplasia to age-matched controls. They found that in both neoplastic conditions, there was a significant reduction in the number and size of BMAds, as well as changes in the expression of certain proteins. They also observed an unusual morphology in BMAds from a patient with metastasis of a malignant glioma. These findings suggest that BMAds undergo significant changes in neoplastic conditions, which may have an impact on the microenvironment and could potentially be used as markers for diagnosis and understanding of these diseases. Further research is needed to understand the mechanisms behind these changes and their clinical implications.

Turner et al. studied the relationship between thermoregulation, BMAT and the hormone leptin in mice. They found that higher housing temperatures led to higher levels of BMAT, white adipose tissue mass and serum leptin in mice and that the administration of leptin led to lower BMAT and higher bone formation in the distal femur metaphysis of mice. However, these effects were not observed in pair-fed mice. They also found that increased housing temperature led to an increase in BMAT in both wild type and leptin-deficient mice, but this increase was attenuated in leptin-deficient mice treated with leptin. These results suggest that both increased housing temperature and increased leptin have independent but opposing effects on BMAT in mice.

Avilkina et al. used a mouse model of anorexia to study the effects of different levels of weight loss on bone density and the expression of the NAD-dependent protein deacetylase Sirtuin 1 (Sirt1) in BMSCs. They found that more severe and prolonged weight loss was associated with decreased bone density and decreased Sirt1 expression in the bone marrow. They also found that Sirt1 expression may influence the differentiation of bone marrow cells into either fat or bone cells, which could further affect bone density. This study suggests that the severity and duration of energy deficits related to anorexia can have important effects on bone density and Sirt1 expression in the bone marrow.

## Conclusions and perspective

This Research Topic presents the latest research and comprehensive reviews on the origins, pathology and systemic physiology of BMAT. It includes conference reports, best practices and insights from a community of scientists dedicated to advancing research in this field. The peer review process and membership of the Bone Marrow Adiposity Society contribute to the rigor of this research. The Research Topic highlights the growing interest of research in BMAT function and reveals the importance of future studies aimed at understanding the developmental, homeostatic and endocrinological intricacies of BMAT that impact metabolic disorders, bone health and beyond.

## Author contributions

JH prepared the first draft and final manuscript; SB-C and AW edited the manuscript. All authors contributed to the article and approved the submitted version.

